# Rational/antiemotional behaviors in interpersonal relationships and the functional prognosis of patients with rheumatoid arthritis: a Japanese multicenter, longitudinal study

**DOI:** 10.1186/1751-0759-8-8

**Published:** 2014-02-24

**Authors:** Jun Nagano, Takako Morita, Koji Taneichi, Shohei Nagaoka, Sadanobu Katsube, Tomiaki Asai, Masao Yukioka, Kiyoshi Takasugi, Masakazu Kondo, Yasuro Nishibayashi

**Affiliations:** 1 Faculty of Arts and Science, Kyushu University, 6-1 Kasuga Park, Kasuga, Fukuoka 816-8580, Japan; 2 Department of Preventive Medicine, Kyushu University Graduate School of Medical Sciences, Fukuoka, Japan; 3 Hiroshima University Graduate School of Biomedical & Health Sciences, Hiroshima, Japan; 4 Taneichi Rheumatism Clinic, Sapporo, Japan; 5 Yokohama Minami Kyousai Hospital, Yokohama, Japan; 6 Hot Spring of Rehabilitation Nakaizu Hospital, Izu, Japan; 7 Asai Rheumatism and Orthopedics Clinic, Nagoya, Japan; 8 Yukioka Hospital, Osaka, Japan; 9 Dohgo Spa Hospital, Matsuyama, Japan; 10 Kondo Rheumatism and Orthopedics Clinic, Fukuoka, Japan; 11 Gratia Hospital, Minoh, Japan

**Keywords:** Rheumatoid arthritis, Psychological stress, Personality, Emotions, Function, Prospective studies

## Abstract

**Background:**

The repression of negative emotions is a personality factor that received considerable attention in the 1950-60s as being relevant to the onset and course of rheumatoid arthritis (RA). Despite subsequent, repeated criticisms of the cross-sectional nature of the earlier studies, even to date few prospective studies have been reported on this issue. This multicenter study prospectively examined if “rational and antiemotional” behavior (antiemotionality), characterized by an extreme tendency to suppress emotional behaviors and to rationalize negative experiences in conflicting interpersonal situations, is associated with the functional prognosis of patients with RA.

**Methods:**

532 patients with RA who regularly visited one of eight hospitals/clinics in Japan in 2000 were recruited for study. All completed a self-administered baseline questionnaire about lifestyle and psychosocial factors including antiemotionality. Two years after, 460 (mean age, 56.1 years; 54 men and 406 women) of 471 patients who continued to visit the clinics agreed to take the follow-up questionnaire. The functional status of the patients was evaluated by rheumatologists based on the ACR classification system.

**Results:**

A multiple logistic regression model that included baseline demographic, disease activity/severity-related, therapeutic, and socioeconomic factors as covariates found a tendency toward higher antiemotionality to be related to poorer functional status at follow-up. This relationship was not explained by lifestyle factors.

**Conclusions:**

Antiemotionality may be a prognostic factor for the functional status of patients with RA. This finding sheds light on a seemingly forgotten issue in the care of patients with RA.

## Background

Rheumatoid arthritis (RA) is an autoimmune disease characterized by chronic systemic inflammation that mainly affects joints, which causes a loss of physical functioning. It can lead to severe systemic dysfunction and a premature death [1]. Physicians and patients agree that psychosocial stress (stress) in daily life can affect the inflammatory and functional status of patients with RA, and some studies have supported this notion [2-4]. Recent advancement in psychoneuroimmunology has helped clarify the mechanisms by which stress affects the disease through interaction between the endocrine, nervous, and immune systems [5-7].

Stress, individual internal experience, can be affected not only by external stressors, but also by interaction between stressors and individual emotional, cognitive, and behavioral responses to them [4]. When a person responds to stressors with a pattern that is repeated on a frequent basis, such a response pattern can be understood within the context of personality. It is difficult for an individual to control external stressors, but it is possible to alter the properties of personality and response style to stressors, and in turn to modify the effects of stressors to the RA disease status. For centuries, clinicians have been impressed by the role of psychosocial factors on RA, and in the 1950s and 1960s, RA came to be viewed as one of the classical psychosomatic diseases [8,9]. Particular personality factors that possibly relate to the onset and course of RA have received considerable attention [10], and a personality characterized by an inability to express aggressive feelings, e.g., “contained hostility” [11] was the one on which many researchers agreed [12]. The nature of these earlier studies included a cross-sectional design, and they were repeatedly criticized, with reviewers concurring on the necessity for prospective studies that allow better causal interpretations [5,9,13,14]. A “neurotic” personality (anxious and depressed), another hypothesis drawn from some earlier cross-sectional studies that used the Minnesota Multiphasic Personality Inventory, was later denied by longitudinal studies [15,16] and re-interpreted as a result rather than a cause of this chronic disease [17]. To the authors’ knowledge, however, even to date few prospective studies have reported on the classical issue of the repression of negative, especially aggressive, emotions as a personality factor associated with the onset/course of RA.

Grossarth-Maticek and colleagues hypothesized that the “rationality and antiemotionality” or “type 5” behavior is a risk factor for the onset of chronic diseases including cancer and RA [18-20]. The rational-antiemotional behavior (antiemotionality) is characterized by an extreme tendency to suppress emotional behaviors and to rationalize negative experiences in conflicting interpersonal situations. Thus, this behavioral pattern largely fits the concept of the repression of negative emotions, whereas it clearly differs from the concept of neurotic personality. It was found, in a cohort study begun in the 1970s, that a high proportion of persons with a highly typical antiemotionality later developed RA [20]. Moreover, it was shown that “Autonomy Training”, a psychotherapeutic intervention method, could alter such a tendency in the direction of healthier behaviors, leading to better physical outcomes [21-23]. A recent study showed that antiemotionality may also be a prognostic factor for lung cancer patients [24]. The present multicenter cohort study examined if antiemotionality is associated with a poorer prognosis for the functional status of patients with RA.

## Methods

### Subjects

The baseline data for the present study was taken from a multi-center cohort survey of Japanese patients with RA carried out in 2000. It was part of a study supported by the Ministry of Health and Welfare, Japan entitled “Assessment and improvement of the system for interdisciplinary medical services for RA (AISIMS)” [25]. Rheumatologists, one working at each of the 12 hospitals/clinics that participated in the program, invited patients with RA who regularly visited their hospitals/clinics to cooperate with the AISIMS cohort study. Patient eligibility included age between 20 and 79 years, the ability to answer a self-administered questionnaire without assistance, and functional status of class 3 or better by the criteria of the American College of Rheumatology (ACR) (see below) [26]. The baseline survey consisted of a self-administered questionnaire for the patients and a clinical data sheet completed by their rheumatologist. The questionnaire inquired about a variety of factors including activity of daily living, quality of life, and lifestyle (smoking, alcohol-drinking, physical exercise, sleep, diet, etc.) and about psychosocial factors (major life events, stress/personality, etc.). The items for the clinical data sheet included factors relevant to the disease status of RA, such as progression of arthritis, functional status, extra-articular complications, and factors associated with medical treatment.

In 2002, two years after the baseline survey, eight of the 12 hospitals/clinics participated in the follow-up survey, with the 23 rheumatologists who were working at these eight hospitals/clinics completing the same clinical data sheet as that used for the baseline survey and handing each patient participant a follow-up questionnaire with items selected from the baseline questionnaire. The follow-up survey was completed before 2003, when the first biologic agent (infliximab) became available in Japan for the treatment of patients with RA [27], because an expected high effectiveness of the agents [1] could overshadow the associations of lifestyle and psychosocial factors with the prognosis of patients with RA and lower the statistical power to detect them. The patients who agreed to cooperate with the follow-up survey sent their completed questionnaires to a central office located at the Department of Preventive Medicine, Kyushu University Graduate School of Medical Sciences. The rheumatologists who evaluated the patients’ clinical status were blinded from the patients’ answers for both the baseline and follow-up questionnaires.

### Measurements

The rheumatologists assessed their patients’ functional status based on the criteria for classification of functional status in RA defined by ACR (ACR class), which classifies patients with RA into one of four classes as follows: Class I: completely able to perform usual activities of daily living (self-care, vocational, and avocational); Class II: able to perform usual self-care and vocational activities, but limited in avocational activities; Class III: able to perform usual self-care activities, but limited in vocational and avocational activities; and Class IV: limited in ability to perform usual self-care, vocational, and avocational activities [26]. They also assessed the status of joint damage based on the classification by Steinbrocker et al. (joint stage: Stage I, Early; Stage II, Moderate; Stage III, Severe; and Stage IV, Terminal) [28]; specified afflicted joints defined as those with either tenderness, swelling, or deformity; and listed extra-articular complications using the following options: cervical myelopathy, cardiac/pericardial manifestations, pulmonary/pleural manifestations, ocular manifestations, peripheral nervous manifestations, hematological manifestations, and others.

Personality factors including antiemotionality were assessed using the “Stress Inventory (SI)” [29,30]. SI is a self-administered questionnaire consisting of 45 items that was developed to assess response styles to stressors principally related to an interpersonal relationship or to chronic stress posed by the response style, that are relevant to chronic diseases [31-33]. Of the 12 scales constituting the SI, the “rationalizing conflicts/frustrations (RCF)” scale measures an extreme tendency to rationalize one’s interpersonal situations accompanied by conflicts or frustrations, and was developed to represent antiemotionality. This scale consists of five questions, such as “do you under all circumstances try to control your reasoning and avoid, as much as possible, being emotional?” (Cronbach α = 0.78). The answers receive a 1 to 6 rating, where 1 and 6 respectively correspond to “yes” and “no” or to “almost always” and “rarely”, and the scores are averaged for the scale score, thus a higher score represents a higher tendency.

### Analysis

The ACR class at follow-up was dichotomized (poorer function, Class 3 or 4 vs. better function, Class 1 or 2) and used as the outcome variable representing the functional prognosis of the patients with RA. The association between antiemotionality and the functional prognosis was examined using a multiple logistic regression model that included the dichotomized ACR class at follow-up as a dependent variable, the RCF score (1–6) as an independent variable, and baseline ACR class (Class I, II, III, IV = 1, 2, 3, 4, respectively) as a covariable. The association was then examined after controlling for known or potential confounding factors including sex; age; factors relevant to disease progression or activity: joint stage (Stage I, II, III, IV = 1, 2, 3, 4, respectively), afflicted joints count (1–45), number of extra-articular complications (0–5), C-reactive protein with log-transformation; medical treatments: methotrexate, corticosteroids, other DMARDs (yes = 1, no = 0); and socioeconomic status: education level (junior high school = 1, high school = 2, junior college = 3, college = 4). Finally, potential intervening effects of lifestyle factors were examined by adding the following variables into this multivariate model: smoking (current smoker = 1, non-/ex-smoker = 0), alcohol drinking (current drinker = 1, non-/ex-drinker = 2), exercise habit (none = 0, 1–3 times/w = 1, 4+ times/w = 2), sleep habit (7–8 hrs/night =1, others = 0), green-yellow vegetable intake (not eat = 1, once/w = 2, 2-4/w = 3, 5-6/w = 4, every day = 5) and fruit intake (same as green-yellow vegetables).

## Results

At the time of the follow-up survey, seven of the 532 patients had died, 13 were visiting another hospital/clinic, and 33 had quit visiting for unknown reasons. The doctors in charge handed the follow-up questionnaire to the 479 patients who continued visiting, and 460 of them sent a completed questionnaire to the central office. Table 1 summarizes the demographics and baseline characteristics of the subjects available for analysis. The characteristics of the patients who were lost to follow-up (visiting another hospital/clinic or having quit visiting for unknown reasons, N = 46) were not statistically different from those of the subjects included in the analysis (see Additional file 1: Table A1). Table 2 shows the baseline characteristics of the subjects according to the degree of antiemotionality. Greater antiemotionality was associated with female sex, older age, and greater number of afflicted joints.

**Table 1:** Demographic and baseline clinical characteristics of patients with rheumatoid arthritis (N = 460)

**Characteristics**	**N**	**(%)**
Age, yrs, mean (SD)	56.1	(9.6)
Female	406	(88.3)
Education completed		
Junior high school	114	(24.8)
High school	251	(54.6)
Junior college or higher	95	(20.6)
Disease duration, yrs, mean (SD)	11.4	(9.5)
ACR class		
1	76	(16.5)
2	322	(70.0)
3	62	(13.5)
Joint stage^*^		
1	55	(12.0)
2	98	(21.3)
3	113	(24.6)
4	194	(42.2)
Afflicted joint^*^ count (0–49), mean (SD)	10.0	(8.8)
No. of extra-articular complications^*^		
0	351	(76.3)
1	83	(18.0)
2+	26	(5.7)
C-reactive protein, mg/dl, mean (SD)	1.63	(2.11)
Medical treatments^†^		
Methotrexate use	194	(42.2)
Corticosteroids use	205	(44.6)
Other DMARDs use	246	(53.5)

SD: standard deviation, ACR: American College of Rheumatology, DMARDs: disease modifying anti-rheumatic drugs. ^*^See text for explanation. ^†^Based on a yes/no choice. Values are N (%) unless otherwise stated.

**Table 2:** Association at baseline between antiemotionality and the demographic and clinical characteristics of patients with rheumatoid arthritis (N = 460)

**Characteristics**	Antiemotionality (RCF score^*^)			**P**^**†**^
	**Low N = 145**	**Moderate N = 157**	**High N = 158**	
Female	82.8	86.6	95.0	.001
Age, yrs, mean (SD)	54.9 (10.0)	56.0 (10.3)	57.4 (8.5)	.033
Education, college or higher	22.8	20.4	19.0	.85
Duration, yrs, mean (SD)	11.6 (9.8)	9.9 (8.5)	12.7 (10.0)	.41
ACR class > = 3	13.8	11.5	15.2	.75
Joint stage^‡^ > = 3	65.5	66.2	68.4	.45
Afflicted joints^‡^ count, mean (SD)	9.9 (9.4)	9.0 (7.8)	11.1 (9.0)	.028
No. extra-articular complications^‡^ > = 1	23.5	23.0	24.7	.49
CRP, mg/dl, mean (SD)	1.49 (1.87)	1.76 (2.50)	1.63 (1.90)	.12
Methotrexate use	38.6	43.4	44.3	.48
Corticosteroids use	44.8	42.0	46.8	.86
Other DMARDs use	54.5	54.1	51.9	.95

RCF: rationalizing conflicts/frustrations. ACR: American College of Rheumatology, CRP: C-reactive protein, DMARDs: disease modifying anti-rheumatic drugs. ^*^The score of the rationalizing conflicts/frustrations scale of the Stress Inventory; the degrees of antiemotionality, “low”, “moderate”, and “high”, were based on the tertiles of the RCF score, 3.8 and 4.6. ^†^Based on Spearman’s rank correlation. ^‡^See text for explanation. Values are % unless otherwise stated.

**Figure 1:**
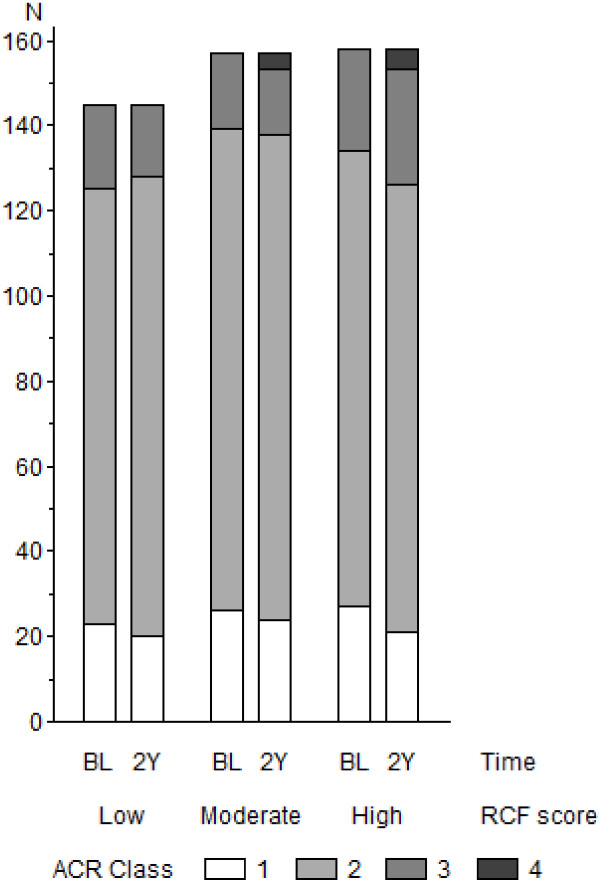
**Changes in the functional status of patients with rheumatoid arthritis according to the degree of antiemotionality.** RCF score: the score of the rationalizing conflicts/frustrations scale of the Stress Inventory; “low”, “moderate” and “high” represent the RCF score of < 3.8, 3.8 to < 4.6, and 4.6+, respectively. ACR: American College of Rheumatology. BL: at the time of the baseline survey, 2Y: two years after the baseline survey.

The ACR class distribution at the follow-up was as follows: Class 1, 65 (14.1%); Class 2, 327 (71.0%); Class 3, 59 (12.8%); and Class 4, 9 (2.0%); thus, global functional status had improved for 56 (12.2%), was unchanged for 328 (71.3%), and had deteriorated for 76 (16.5%) over the two-year study period. Figure 1 shows the change in ACR class over the period according to the RCF score.

Table 3 shows the results of the multiple logistic regression analysis done to examine the association between antiemotionality and the functional prognosis. Greater antiemotionality (a higher RCF score) was associated with a poorer ACR class two-years after the baseline survey in the model that only included baseline ACR class as a covariate (Model 1). When multiple factors relevant to demographics, disease activity/progression, treatments, and socioeconomic status were additionally adjusted, the association between antiemotionality and the functional prognosis did not materially change (Model 2). A one point increment in the baseline RCF score was associated with a nearly 50% higher chance of having a poorer functional status rather than a better status at the follow-up. The possible intervening effect of lifestyle factors, including smoking, alcohol consumption, exercise, sleep hours, green/yellow vegetable intake, and fruit intake, was then examined. Additional adjustment for these factors, however, did not appreciably change the association between antiemotionality and the functional prognosis (OR = 1.60, 95% CI = 1.16-2.22, P = 0.005).

**Table 3:** Multiple logistic regression analysis of the association between antiemotionality at baseline and the functional prognosis (ACR class at follow-up) of patients with rheumatoid arthritis

**Baseline variables**	**Model 1**			**Model 2**		
	**OR**	**(95% CI)**	**P**	**OR**	**(95% CI)**	**P**
Antiemotionality (RCF score^*^)	1.40	(1.05-1.87)	.023	1.47	(1.07-2.00)	.016
ACR class	10.52	(5.87-18.9)	<.001	6.13	(3.17-11.8)	<.001
Female sex				1.92	(0.53-6.98)	.32
Age				1.00	(0.96-1.04)	.99
Joint stage^*^				1.20	(0.80-1.78)	.38
Afflicted joints^*^ count				1.03	(1.00-1.07)	.06
No. extra-articular complications^*^				1.40	(0.96-2.02)	.08
Log. C-reactive protein				1.30	(0.99-1.71)	.06
Methotrexate use				1.39	(0.67-2.88)	.38
Corticosteroids use				0.43	(0.20-0.94)	.034
Other DMARDs use				1.12	(0.54-2.32)	.76
Education level				0.80	(0.52-1.23)	.30

ACR: American College of Rheumatology. OR: odds ratio of a poorer function (Class 3 or 4) vs. a better function (Class 1 or 2) at follow-up. CI: confidence interval. RCF: rationalizing conflicts/frustrations. ^*^See text for explanation.

## Discussion

This Japanese, multicenter, cohort study supports the hypothesis that a strong tendency toward antiemotionality, previously reported as a risk factor for the onset of RA [20], is also associated with the functional prognosis of patients with RA. This is, to the authors’ knowledge, the first prospective study that supports the classical notion that a personality characterized by emotional suppression has a particular connection with the course of RA. The RCF score was differently correlated with specific aspects of negative emotionality in a study of its psychometrical validity [30]. Its positive and negative correlations respectively with “anger-in” and “anger-out”, concepts by Spielberger [34], support earlier cross-sectional findings of the inability to express aggressive feelings associated with a poorer course of this disease [10-12]. That the RCF score was not correlated with trait-anxiety or depression is consistent with the null association between “neurotic” personality and the course of RA in previous longitudinal studies [15,16].

Although this study was of a prospective design, the observed association will need to be further scrutinized for possible confounding effects. Factors representing disease activity, chronic symptoms and functional disability, and medical treatments that potentially affect the central nervous system may have affected the baseline RCF scores. Socioeconomic status, known as a possible prognostic factor for patients with RA [35], may also be correlated with RCF. However, the prospective association of antiemotionality with functional disability at follow-up was independent of all of these baseline factors.

Except for smoking’s exacerbating effect, no lifestyle factor has been established as affecting the disease course of RA [1,36]. However, lifestyle factors should be considered major pathways that potentially link psychosocial variables and physical outcomes. Various factors considered to be generally favorable or unfavorable for health, such as smoking, alcohol-drinking, physical exercise, sleep, and diet were examined for their potential intervening effects, but they did not explain the antiemotionality-prognosis association.

Another pathway to be considered is the psycho-physiological system. The hypothalamus is the center of the autonomic nervous system (ANS) and of the hypothalamus-pituitary-adrenal (HPA) axis, and it has been progressively elucidated that the ANS, HPA axis, and immune system are closely connected and that they interact with each other [5-7]. When the cerebral cortex, the center of reason, chronically and overly interferes with the limbic system, which is responsible for emotional responses, this may affect the function of the hypothalamus, which is closely connected to the limbic system, and in turn the function of the immune system. Such behaviors by which a person attempts to maintain imperturbability or to understand another person when confronting a frustrating interpersonal situation will be, when appropriately employed, harmless or rather preferable. However, when such behaviors are extremely employed, i.e., negative emotions are always rationalized and their expression is blocked, the emotions that lost outlet may damage the functioning of biological homeostasis. Ishii et al. found that patients with RA who were easily moved to tears as a response to psychological stress showed a better response to treatment and a better general prognosis than those who did not show such emotional responses [37].

This study has several limitations. First, no biomarkers, such as neuroendocrinological or immunological markers were measured that can be related to the above discussion of the psycho-physiological pathway. Second, although the outcome measure used, ACR class, is convenient for assessment and represents the overall functional status of patients with RA, its sensitivity to detect temporal changes of the status is limited [38]. The assessment depends completely on the subjective evaluation of the rheumatologist, while other commonly used scoring systems, such as the ACR core set and the Disease Activity Score, integrate objective markers and subjective evaluations by both doctors and patients [39,40]. Third, the present data only included a small number of male patients, and thus could not address possible sex-related difference in the antiemotionality-prognosis association. Nevertheless, this study adds new findings to the currently insufficient prospective data concerning specific personalities and relevant properties that contribute to the progression of RA. The present results were based on a relatively large sample that was collected at multiple hospitals across Japan and can be generalized to Japanese patients with RA.

## Conclusions

Antiemotionality, a personality trait characterized by strong suppression of becoming emotional and by overly appealing to reason in stressful interpersonal situations, is associated with a poorer functional prognosis for patients with RA. This finding sheds light on a seemingly forgotten issue in the care of patients with RA.

## Abbreviations

ACR: American College of Rheumatology; AISIMS: Assessment and improvement of the system for interdisciplinary medical services for rheumatoid arthritis; ANS: Autonomic nervous system; Antiemotionality: Rational and antiemotional behavior; DMARD: Disease-modifying antirheumatic drug; HPA: Hypothalamus-pituitary-adrenal; RA: Rheumatoid arthritis; RCF: Rationalizing conflicts/frustrations; SI: The Stress Inventory.

## Competing interests

The authors declare that they have no competing interests.

## Authors’ contribution

YN, TM, and JN conceived of the study. All authors designed the study and collected the data. JN and TM analyzed the data. JN prepared the draft of the manuscript, and MY and YN helped draft the manuscript. All authors read and approved the final manuscript.

## Supplementary Material

Additional file 1: Table A1 Demographic and baseline clinical characteristics of patients with rheumatoid arthritis who were lost to follow-up (N = 46). Click here for file
