# Supplementary material for: Rational/antiemotional behaviors in interpersonal relationships and the functional prognosis of patients with rheumatoid arthritis: a Japanese multicenter, longitudinal study
Source: Biopsychosoc Med. 2014 Feb 24;8:8. doi: 10.1186/1751-0759-8-8 (PMC3941968; doi:10.1186/1751-0759-8-8)
Supplement: Additional file 1: Table A1 — Demographic and baseline clinical characteristics of patients with rheumatoid arthritis who were lost to follow-up (N = 46). [file 1751-0759-8-8-S1.pdf]

Appendix

Table A1. Demographic and baseline clinical characteristics of patients with rheumatoid arthritis who were lost to follow-up (N = 46)

Characteristics	N	(%)
Age, yrs, mean (SD) *	55.9	(11.2)
Female†	41	(89.1)
Education completed‡		
Junior high school	9	(19.6)
High school	23	(50.0)
Junior college or higher	14	(30.4)
Disease duration§, yrs, mean (SD)	11.5	(10.4)
ACR class‡		
1	13	(28.3)
2	23	(50.0)
3	10	(21.7)
Joint stage‡		
1	7	(15.2)
2	11	(23.9)
3	8	(17.4)
4	20	(43.5)
Afflicted joint* count (0-49), mean (SD)	12.1	(10.4)
No. of extra-articular complications§		
0	42	(91.3)
1	2	(4.4)
2+	2	(4.4)
C-reactive protein§, mg/dl, mean (SD)	1.88	(1.69)
Medical treatments		
Methotrexate use†	16	(34.8)
Corticosteroids use†	18	(39.1)
Other DMARDs use†	25	(54.4)

SD: standard deviation, ACR: American College of Rheumatology, DMARDs: disease modifying anti-rheumatic drugs. *See text for explanation. Values are N (%) unless otherwise stated. No statistical difference was found in the distribution of the participants lost to study and those for whom data was available for analysis (N = 460, see Table 1) (Student's t-test*, chi-square test†, Mantel-Haenszel chi-square test‡, or the Wilcoxon rank sum test§).
